# Facilitators and Barriers to Accessing Mental Health Care for Older Adults Who Are Primary Care Users in Chile: A Focus Group Study

**DOI:** 10.3390/healthcare14101341

**Published:** 2026-05-14

**Authors:** Ximena Moreno, Paula García, Martín Huerta

**Affiliations:** 1 Instituto de Salud Pública, Universidad Andrés Bello, Santiago 7591538, Chile; 2 Independent Researcher, Santiago 8390442, Chile; paulagarciayevenes@gmail.com (P.G.); martinhterapias@gmail.com (M.H.)

**Keywords:** older adults, mental health, primary care, integrated care, social determinants of health, qualitative study, aging

## Abstract

**Background/Objectives**: Mental health conditions are highly prevalent among older adults, and they are associated with adverse health trajectories. There is a considerable gap in mental health care access in this group. It is important to examine factors that affect mental health care access from the perspective of older adults. This study aimed to understand factors that affect mental health care access from the perspective of older adults who are primary care users in Chile. **Methods**: Sixty-two people aged 62 to 88 participated in eight focus groups (5–11 participants per group) conducted in community settings in Santiago. The focus groups were audio-recorded and transcribed verbatim. Framework analysis was carried out. **Results**: Facilitators and barriers for accessing mental health care were organized around five themes: (1) Life transitions in older age, (2) Representations of mental health in older age, (3) Pertinence of mental health programs to older adults’ needs, (4) Organization of health services, (5) Health providers’ skills. **Conclusions**: From a primary care policy perspective, it is critical to move beyond mental health program availability and implement an integrated care approach with adequate funding, specialized training, and person-centered protocols. Challenges associated with life transitions and specific needs expressed by older adults should be considered in person-centered care plans.

## 1. Introduction

Global population aging is accelerating, with the number of people aged 65 or older projected to more than double over the next three decades [1]. Common mental health disorders are highly prevalent among older adults, affecting 19.2% and 16.5% of this group of the population, respectively [2]. Longitudinal studies suggest a bidirectional relationship between mental and physical health in older age [3,4], with mental health conditions increasing the risk of adverse outcomes, including cardiovascular events, functional decline, and premature mortality [5,6]. The distribution of mental health problems is unequal and shaped by social determinants, such as education, income, social networks, housing, neighborhood conditions, access to health care, and family and personal characteristics [7]. Social determinants interact in complex, context-specific ways [8], influencing mental health through the interplay of social positions across the life course [9]. Furthermore, social determinants have a cumulative effect on mental health. Older adults with a history of socioeconomic or material disadvantages during their childhood are more likely to experience mental health problems and lower wellbeing throughout adulthood and in older age [10,11].

Access to mental health care is constrained by social determinants, with a lower concentration of primary care providers in disadvantaged areas [10], unequal access to non-medical mental health services [11], and reduced access to mental health care among older adults with depression and anxiety [12,13]. Previous studies suggest that barriers to mental health care for older adults partly stem from primary care services organization, including issues such as high workload that limits time to build patient rapport, fragmented care approaches, or insufficient access and communication with specialty mental health services [14,15]. Limited training and experience among health providers can hinder the recognition of signs of depression in older adults, as specific knowledge and communication skills are needed to elicit and interpret relevant diagnostic information [16,17,18]. Prior research indicates that health providers often misinterpret depression symptoms in older adults as normal aging or as expressions of physical distress [12,19]. Likewise, primary care physicians are less likely to ask about depression, suicide risk, or to discuss mental health topics with older patients [20]. Additionally, despite evidence supporting the effectiveness of psychotherapy to treat depression and anxiety in older adults [21,22], this age group is less likely to be referred to such services [23]. This is consistent with ageist beliefs among some mental health providers, who perceive older adults as less capable of developing therapeutic relationships compared to younger patients with similar symptoms [24]. With the exception of Colombian study [16], this literature reflects evidence from the Global North.

Previous studies have examined facilitators and barriers to mental health seeking from the perspective of older adults. Mental health stigma acts as a barrier for help-seeking and symptom reporting among older adults [25,26,27]. Furthermore, this age group may experience double stigmatization, resulting from the intersection of ageism and mental health stigma [24,28]. Likewise, attitudinal barriers including concerns about negative reactions from family and friends, alongside the possibility of being perceived as a burden, have also been identified [29]. Limited knowledge of available services represents a barrier to help-seeking among older adults with depression [28]. Additionally, some studies report that older adults cite cost as a barrier to accessing mental health care [26,27]. Conversely, facilitators include mental health literacy, positive previous experience with mental health providers, perceived trust and support from clinicians, and informal support networks, such as family and friends [26,28,30]. A large-scale qualitative study identified provider-related facilitators that were valued by older adults, including patient-centered communication, rapport building, accessible language, and non-judgmental care [31].

Despite having one of the highest life expectancies at birth (79.7 years) in Latin America [32], nearly 30% of the general population of older adults in Chile has moderate or severe depressive symptoms, with a higher prevalence observed among women [33]. Additionally, older women spend a greater proportion of their remaining lifespan with depression, compared to men of the same age [34]. The diagnostic gap for depression in older adults is considerable, with nearly 70% of cases undetected in the general population [33]. According to the Pan American Health Organization [35], nearly 90% of older adults are users of primary care in Chile. At this level, the Annual Preventive Medical Evaluation of the Older Adult, designed to identify people at risk of dependency or with dependency, includes a depression screening [36]. However, a previous study in Chile estimated that 64% of older adults who were primary care users with moderate to severe depressive symptoms had not been diagnosed [37]. A national program for the detection and treatment of depression has been implemented in all primary care centers in Chile [38]. Additionally, depression is included in the Program of Explicit Health Guarantees, to provide timely access, quality, and financially protected care for people aged 15 years and older diagnosed with depression [39]. Nevertheless, no specific guidelines for the detection and treatment of depression in older adults are considered in these strategies.

Given that existing mental health programs often fail to address the specific needs of older adults, the Intersectoral Committee on Mental Health in Older Adults has emphasized the need to design a tailored mental health strategy for older people and to define specific criteria to monitor and treat common mental health conditions in this group [40]. Incorporating older adults’ perspectives on mental health care access is crucial for designing pertinent and effective mental health interventions. Therefore, this study aimed to understand factors that affect mental health care access from the perspective of older adults who are primary care users in Chile.

## 2. Materials and Methods

This study follows the COREQ guidelines for reporting qualitative research [41]. Specific sections corresponding to each item are described in Supplementary File S1.

### 2.1. Study Design

This study presents results from a focus groups study conducted in Santiago, Chile, between 2024 and 2025, as part of a mixed-methods project initiated in 2023. The mixed-method study aimed at exploring barriers and facilitators for accessing diagnosis and treatment for depression among older adults in primary care in Chile [42,43]. The qualitative phase of this study explored older adults’ perspectives on facilitators and barriers to mental health care access. The scope of this phase was broadened to include general mental health concerns, allowing for the inclusion of other experiences of mental distress relevant to older adults. In the quantitative phase, participants identified depression as a primary mental health problem covered by health programs, but they also cited anxiety and broader issues such as social isolation or family problems. This comprehensive focus aimed to enrich the discussion about the facilitators or barriers that older adults experience when accessing these services.

### 2.2. Setting and Participants

The study participants were older adults enrolled in primary care in two municipalities of the northern area of Santiago, Chile. These municipalities are classified as having medium social vulnerability by the Chilean Ministry of Social Development [44], with nearly one fifth of inhabitants experiencing multidimensional poverty [45]. For this qualitative study, 62 people were recruited purposively from a sample of 253 community-dwelling older adults who had been recruited from primary care or community groups for older adults during the initial quantitative stage of the mixed methods project. People unable to leave home or to participate in an interview due to health conditions were excluded [42,43]. As a result of these eligibility criteria and the sampling strategy, participants differed from other older community-dwelling older adults who experience severe health problems, mobility limitations, and higher social isolation. During recruitment, participants signed an informed consent form and participated in a face-to-face structured interview, as part of the quantitative phase. At the end of the interview, participants were asked for consent to be contacted during the following months to participate in a focus group, with an acceptance rate of 96%. Ten participants declined to be contacted for the qualitative phase, including nine women. No other sociodemographic or health differences were observed, compared to the rest of the sample. The main reason for declining was anticipated unavailability due to health reasons or travel.

Based on the literature consistently reporting gender differences in mental health care access for older adults [12,33], participants were purposively contacted to form separate male and female groups, to explore gendered perspectives and experiences about mental health care access. As previous studies suggest, homogeneous groups foster open dialogue regarding shared gender-related experiences [46]. Thematic saturation on gendered experiences was reached after conducting three focus groups with men and three groups with women. These themes highlighted how gender roles shaped life transitions and close relationships at this age. Nevertheless, it was considered important to explore emerging themes, such as organization of health services or participation in community groups, that were not discussed in depth within gender homogeneous groups. Two additional mixed-gender focus groups were conducted to gain a deeper understanding of the facilitators and barriers to mental health care access, exploring shared experiences among men and women. Participants in each group were heterogeneous in age, level of education, marital status, and depressive symptoms.

To contact focus groups participants, the original database was stratified by gender and ordered by recruitment date. Participants were contacted via telephone, based on the chronological order of initial recruitment. People reachable by phone were asked to confirm their willingness and availability to take part in a focus group. Most contacted participants agreed to take part, but some declined due to scheduling conflicts, travel or health reasons. Participants available for a future focus group were re-contacted.

### 2.3. Data Collection

Focus groups were selected to foster open dialogues, allowing participants to express and discuss diverse perspectives on topics related to the research question [47]. The flow of the dialogue, including comments, explanations, and disagreements, enriches the understanding of which topics mobilize group participants, and how they construct meanings [48]. Focus groups were conducted in community settings between May 2024 and January 2025. To ensure participants’ comfort and privacy, a room with adequate ventilation, temperature, and privacy was used. Light refreshments were offered during the sessions. Two researchers participated in each focus group, a psychologist (XM or MH) and a sociologist (PG). One of them acted as moderator, introducing the purpose of the study, ethical considerations and ground rules, while guiding the discussion. Ground rules established a safe environment and promoted balanced participation. When one or more participants dominated the conversation, the moderator encouraged participation of the rest of the group, emphasizing that diverse perspectives were important to enriching the discussion. The other facilitator received participants, provided refreshments, audio-recorded the session, and took field notes. These notes were used to document the emotional tone of discussions, identifying topics that mobilized the conversation and fostered consensus or disagreement. Furthermore, they provided insights into which themes needed further exploration and highlighted emergent topics to include in successive groups. Two researchers, MH and PG, had previously interviewed participants during the quantitative phase. To build on established rapport, they act as moderators in focus groups. XM assisted and took notes in three sessions.

The research team designed guiding questions (Table 1), based on the literature about facilitators and barriers to accessing mental health care, as described in the Introduction. Based on field notes and analysis, this guideline was reviewed and refined after each focus group to include questions on emerging themes in the following groups, such as the use of digital technologies. Question order remained flexible, allowing for adaptation if a theme was spontaneously addressed earlier. Additional questions were used to explore specific themes more in depth. Focus groups lasted between 80 and 100 min and were audio-recorded. Information regarding local mental health services was available from facilitators, but no participant experienced distress during the sessions.

### 2.4. Data Analysis

Focus groups were transcribed verbatim by a research assistant, with participants’ names replaced by labels (participant 1, participant 2, etc.). The authors employed framework analysis, a codebook approach of thematic analysis to conduct deductive analysis based on predefined themes, but having the flexibility to develop iteratively new themes, as the analytical process progressed [49]. Framework analysis involves five phases: familiarization, framework development, indexing, charting, and mapping and interpretation [50].

As an initial step, after each focus group, all the researchers immersed themselves in the data by reading transcripts and taking notes, which informed subsequent coding and team discussions. The initial framework, based on prior research about barriers and facilitators, was used to design the focus group questions, covering representations of older adults, mental health stigma, information regarding mental health care, previous mental health care experience, primary care organization, trust in care providers, and social support. This strategy allowed the research team to organize the information into broad categories, which were refined by independently pilot-coding the first transcript and comparing, discussing and aligning results. Reflexive individual notes and data quotes were used to solve discrepancies and reach consensus on code definitions, data re-coding or development of new codes. Emerging codes focused on the pertinence of health programs, responsibility for mental health, and expectations about mental health providers. Each researcher coded at least two focus groups. Team meetings were held to review codes and individual notes, allowing for further refinement of the framework.

In the indexing phase, transcripts were coded into one or several categories of the framework. Data was charted and summarized by category and by group, including key quotations. The team collaboratively mapped and interpreted the data. Thematic saturation was achieved after the eighth focus group, with no new codes or themes emerging from the analysis [51].

MAXQDA 24 was used to support data organization, coding process, and note-taking during the analysis.

### 2.5. Research Team and Rigor

The research team was formed by a social psychologist (XM), with postgraduate degrees in public health, and experience in community-level field research. She currently works as an academic, specializing in aging, mental health, and social inequalities. Another member of the team (PG) is a sociologist, specializing in community research and participatory methodologies, including forum theater and participatory action research. She has extensive experience in facilitating community-based mental health initiatives with participation of older adults. The other member of the team is a clinical psychologist (MH), trained in the community model of primary care and research methodologies. He has clinical experience working with older adults in primary care, and he has participated in research projects focused on health and aging. Participants had previously met PG or MH during a structured interview for the quantitative phase of the mixed-methods research, one year earlier. This allowed researchers to familiarize themselves with the context and build rapport during the focus groups.

Credibility was enhanced through reflexivity and researcher triangulation [52]. Reflexivity involved an iterative process of individual and team-based reflections about how researchers’ personal background influenced data interpretation. Researcher triangulation, through the discussion of each researcher’s interpretations of the data, provided richer insights into older adults’ perspectives on mental health and access to care. To support transferability, details about the context where the research was conducted are provided in the setting section, and participants’ characteristics and quotes are used to illustrate the results. To enhance confirmability, individual notes were taken after familiarization with the data and during the coding process, and they were used during team meetings to discuss and guide decisions about recoding data or creating new codes.

### 2.6. Ethical Considerations

The protocol of the study was reviewed and approved by the Ethics Committee of the North Metropolitan Health Service. Before taking part in the study, participants received information about the background, aims, and activities considered in the study. All participants signed an informed consent. This study adheres to the Declaration of Helsinki [53].

## 3. Results

A total of 62 people (60% women) participated in eight focus groups, with group sizes ranging from 5 to 11 per group. As shown in Table 2, participants were aged 62–88, and they were heterogeneous in education level, marital status, and depressive symptoms.

The information analyzed by the research team was organized into five themes, involving aspects that could facilitate or limit access to mental health care: life transitions in older age, representations of mental health in older age, pertinence of mental health programs, organization of health services, and health providers’ skills.

### 3.1. Life Transitions in Older Age

This theme describes how gendered experiences and expectations during life transitions influence mental health status and shape mental health help seeking. Its interaction with other themes is described in the following sections.

Transitions during older adulthood were identified by participants as a source of distress that could have a negative mental health impact. In the case of men, the impact of retirement was described as a profound loss of role, routine, and personal meaning: “It is quite true, what my dear friend says, about the change you experience when you retire. I suffered at least six months. I didn’t feel comfortable at home. What to do? How bad! I’m a sort of handyman, so I started fixing things. But then, there were no more things to fix, so what shall I do?” (Focus group 2, male participant).

Among women, several described the empty nest transition, noting that changes in the quality of their relationships with children contributed to feelings of loneliness and perceived loss of significance for their children: “Because loneliness is a theme that makes us feel that we need something… a time comes when children, with their duties and everything... it is not that they desert you, but they have many things that maybe, for them, are indispensable. Then you are left behind… after you have given everything that you could, and children inadvertently somehow put you aside” (Focus group 1, female participant).

As described above, role loss was observed across genders. However, men emphasized a loss of participation in the public sphere, whereas women focused on the family sphere and how the quality of relationships with children failed to meet their expectations.

Apart from those feelings and meanings associated with life transitions, a gender-shaped imperative of maintaining abilities was observed. Women with life trajectories focused on domestic roles, including household management, childcare and caring for older adults, were concerned with preserving their ability to care for others: “No, my mom has medical checks at the hospital, and she has had a quite good treatment, and we also have been in a very catastrophic environment. What I perceive of the question is that in short time I will be very complicated with poor sleep, because I have sleep issues. So, since I must assist my mother at night, it doesn’t work very well the medication aspect. So, I need to go to a psychiatrist to see what to do with my medication… it has been complex here, I mean, I care a person with dependency problems” (Focus groups 7, female participant). Motivated by caregiving responsibilities, this participant was willing to seek professional help to improve her sleep quality, recognizing that maintaining her mental health was important to care for her mother.

For men, not depending on others also referred to the capacity to make their own daily life decisions: “I have three daughters… The three of them compete for me, every week—dad, daddy, when are you coming with me? Why are you alone?—If I live, it is because I’m alone. Thanks to that, I’ll keep on living. And I hope to never depend on any of you, I say to them. Because one must have that mentality.” (Focus group 2, male participant). While this participant values autonomy, this view could hinder the identification of mental health needs and act as a barrier for mental health care seeking, if they are associated with losing independence.

### 3.2. Theme 2. Representations of Mental Health in Older Age

When describing the meaning of mental health in older age, participants frequently referred to mental health problems and symptoms, in some cases normalizing them as an aspect of aging: “Yes, I wanted to put forward the subject of depression that occurs as one gets older. It seems like a natural part, when you see you’re getting alone a bit due to age. It doesn’t mean that you’re not surrounded by people, you’re getting alone, you’re becoming introverted…” (Focus group 4, male participant). These beliefs could hinder the ability to recognize mental health needs and to express mental health concerns, acting as barriers for mental health care access.

In some discussions, participants highlighted individual traits and attitudes to face problems as central to maintaining mental health, while mental health problems were viewed as lack of personal strength to overcome life crises: “Ehm… I believe that it depends more than anything on the character of each person. Because when there are strong characters, it is easier to dominate situations such as depressions.” (Focus group 8, female participant). Although life crises were gendered, perspectives on individual characteristics and personal resilience were shared across both males and female groups: “To me, depression does not exist. I am my own psychologist. Yes? In specific cases, I have literature that I read, I read everything. And my behavior with my friends, I’m an extroverted guy, right? So, I don’t have that. And I know that anything, you must trace it in your mind, it will work since today.” (Focus group 4, male participant). These views reinforce stigmatized views about mental health distress and care seeking among older adults, by equating them with negative personal characteristics.

In contrast, other participants acknowledged the utility of seeking mental health care: “It happened to me once, I don’t know if it was depression… And I said, I cannot deal with this alone. Because I didn’t want to get up from bed, I didn’t want to do my things… And I said: I can’t, I’ll go to primary care. And I take refuge in my primary care. I have found good professionals.” (Focus group 1, female participant). This underscores the need to recognize mental health challenges and highlights that previous positive experiences with primary care facilitate mental health care seek in case of distress.

### 3.3. Theme 3. Pertinence of Mental Health Programs to Older Adults’ Needs

Mental health services were valued as important and necessary in some group discussions, but limitations were described in terms of insufficient consideration of the transitions of this life stage and the needs of older adults. This was particularly discussed by women. As described in the theme about life transitions in older age, feelings of loneliness, loss of significance, and the preservation of abilities to care for others were the main concerns expressed by them. In this sense, some female participants highlighted a contrast between programs tailored for women in reproductive age and those focused on older adults, which lacked an age and gender perspective: “… because when we are pregnant, they inform us, they guide us about what we have to do. When the child is born, we know the characteristics of that child. It is this. We must do this when they are young, when they are adults… So, when we get to this stage… I feel we should get advice and more information about this stage, right?” (Focus group 1, female participant). Women’s perception that primary care programs are inadequate may limit mental health care seeking and willingness to discuss mental health concerns. This could also reflect the perception of a deficiency in health provider skills to discuss meaningful psychosocial experiences with them.

It was also noted that older adults’ mental health complaints were frequently normalized, with care limited to severe cases: “Let’s see my case, and forgive me for saying it so, but the problem I have is a family one, but you approach… if you approach the primary care, they say it has no importance at all, so it has to be a more extreme case for they to consider you.” (Focus group 8, male participant). This perception could reflect several factors that contribute to limited access to mental health care for older adults: ageist stereotypes that normalize psychological distress, insufficient mental health training, and a lack of resources in primary care facilities, with limited consultation time that leads to a prioritization of physical health over mental wellbeing.

The tendency to prioritize pharmacological treatments above other approaches to address mental health needs of older adults was critically viewed: “Well, I consider that primary care is in debt with respect to mental health. Unfortunately, yes… I don’t know if there are better techniques or treatments. In the end they only give you pills” (Focus group 3, female participant). Unmet expectations regarding therapeutic strategies for mental health problems can also limit mental health care seeking within primary care.

### 3.4. Theme 4. Organization of Health Services

Barriers for accessing mental health care were described regarding the organization of mental health services. These views were shared across genders. Although primary care centers claimed to prioritize access for older adults to health appointments, participants experienced significant barriers in accessing mental health services: “… the primary care is full of signs saying: older adults have priority. And that, when you get to the front desk you realize it is not like that. That there are no appointments available for psychologist, that older adults have no priority…” (Focus group 5, female participant).

Additionally, problems with the organization of appointments involved extended waiting times and compromised the continuity of care, which participants perceived as exacerbating mental health distress: “Because if they are changing your appointment for this, one day they say to you: come the 5th of January, and they change it to March, it will affect the mental aspect too, I think.” (Focus group 7, male participant).

Participants discussed a perceived rise in primary care patient registration, which affected the availability of mental health providers and shortened the duration of consultations: “The population has grown a lot… the time is not enough to dedicate more time to each specific patient.” (Focus group 3, female participant). This comment highlights that insufficient resources to meet the rising demand affect the quality of mental health care, acting as a barrier to access.

### 3.5. Theme 5. Health Providers’ Skills

This theme describes facilitators and barriers to accessing mental health care, regarding participants’ experiences with mental health providers. The perceived utility of mental health consultations, as discussed by participants, was shaped by the ability of health providers to actively listen to older adults’ concerns and build trusting and ongoing relationships. As expressed by one participant: “In my experience with respect to mental health… with the psychologist with whom I have consultation, he has been good, he has been very… fluent, with him… he has very good interpersonal communication and dialogue, and he gives you the tools for you to progress.” (Focus group 5, female participant).

Another aspect that was positively valued was the ability of health providers to give practical and actionable advice for daily life, as highlighted by a participant: “I had an appointment with a psychologist that I really applaud him, because I went to see him for a very specific situation and he said to me … ‘I’ll be very frank’, but that’s what I need… First time that a person that is so intelligent talks so directly; there are two people that has marked me in my life in that aspect.” (Focus group 8, male participant).

Negative experiences during mental health consultation, including disrespect, lack of empathy and insufficient emotional support, were identified as significant barriers to seeking mental health care. These experiences varied, ranging from perceived lack of professional skills to disrespectful treatment: “I was consulting a psychologist that, while I was talking to her, she fell asleep, yes, she literally fell asleep, and she never took any notes.” (Focus group 8, female participant). “I told her everything and suddenly her phone started ringing, she took it, ‘keep on telling me’, and she was talking by the phone and then… ‘what we were talking about?’, no!, then no, ‘do you know what?, you’ll come in a month’, the month arrives and it happens exactly the same thing…” (Focus group 8, male participant).

## 4. Discussion

The results of this study highlight factors influencing access to mental health care for older adults within the Chilean primary care system. A key contribution of this study is examining how gendered life transitions affect the mental health of this population. Transitions experienced in older age, such as retirement, the empty nest, and increased informal caregiving, are shaped by gendered trajectories, roles and expectations. These transitions influence mental health, access and engagement with mental health care services. As reported by prior research, the gendered impact of retirement on mental health is heterogeneous and deeply influenced by socioeconomic differences, with higher socioeconomic status often associated with improved mental health after retirement, while lower socioeconomic positions are linked to more adverse outcomes [54]. Also, the impact of the empty nest on the wellbeing of older adults is a complex and culturally dependent transition, representing either a role loss with a negative impact on wellbeing, or a role strain relief that expands the possibilities of being socially engaged [55]. In this study, the importance of maintaining functional abilities during these transitions was stressed. These abilities varied from being able to care for others, having physical strength and energy, not depending on others, and having autonomy to control decisions in daily life. While consistent with successful aging models that interpret mental health care seeking as a proactive self-care attitude, these models often reproduce ageism and ableism by disregarding how social determinants affect the possibilities for active and independent aging [56]. Hence, internalized social expectations about maintaining abilities act as barriers to mental health care seeking, which could unequally affect older adults experiencing mental distress. In that regard, previous quantitative analysis of data from these participants showed that older adults who experienced adverse socioeconomic circumstances, including social isolation or a lower level of education, had a less positive attitude towards seeking psychological help [42].

The findings of this study suggest that participants frequently perceived mental health problems as an intrinsic and normal aspect of aging and life transitions. Furthermore, a belief that mental health was solely dependent on personal attitude and the perception of mental health distress as a lack of strength supported stigmatizing views regarding mental health needs. Our results align with international studies indicating that the normalization of mental health problems in older adults, alongside stigmatized views, acts as a barrier to recognizing the need for care and accessing professional support [16,26,28]. Nevertheless, these views were not uniform, with discussions often highlighting broader social context and personal circumstances as key factors influencing mental health, while stressing the importance of seeking professional help. These results suggest that interventions to promote mental health literacy are crucial for reducing internalized stigma and fostering mental health care seeking in older adults [57].

Experiences of using primary care services expressed criticism about the organization of services, which were perceived as poorly tailored to older adults and failing to address life-stage issues. Furthermore, the tendency to prioritize pharmacological treatment for mental health issues was valued negatively. This aligns with existing research reporting that while older adults often prefer and benefit from psychotherapy, they are less likely to access it compared to younger groups of the population [22]. Participants expressed expectations regarding services organization that are consistent with previous findings about barriers to accessing mental health care, including improved appointment availability, higher frequency, and greater continuity of care [14,16,28]. Negative experiences with mental health providers were described by participants, citing dismissed needs, inability to foster trust, and disrespectful attitudes. While positive experiences were attributed more to chance than to the design of primary care programs, specific professional attributes were highly valued. Participants positively assessed providers trained in the life stage and context of older adults who could build trust, make them feel heard, and offer useful advice, reinforcing previously identified facilitators to care [16,26,28,30,58].

Our results have implications for planning programs for older adults in primary care in Chile, particularly those who are frequent users and community engaged. Participants’ views were concordant with an international review regarding the importance of integrated care, highlighting the importance of continuity, coordination, rapid access, and quality [59]. A previous study in Chile evaluated a pilot integrated, patient-centered approach for people with multimorbidity; while it showed promising results, it revealed that mental health has not yet been integrated into patient controls due to inadequate training and a lack of an interdisciplinary approach [60]. An integrated care model for primary care in Chile must be further developed to adequately incorporate mental health components [61]. Implementing these changes requires interrelated strategies, including equitable resource allocation to meet context-specific demands [62], and strengthening organizational capacity regarding program delivery, including the adoption of remote appointment systems [63]. Additionally, training modules for primary care teams are crucial, focusing on technical, attitudinal, and communication skills to address mental health, stigma, ageism, and ableism [18]. Finally, health checkups should incorporate discussions on life transitions to create pertinent and context-specific care plans that bridge primary care with community resources.

Strengths of this study include its focus on an under-researched area in Latin America [16]. By analyzing the perspective of older adults, this work advances understanding of the dimensions affecting mental health care access in the region. Consequently, the results offer evidence-based guidelines for developing context-specific programs and strengthening public health policies aimed at improving the mental health of this population. Several limitations regarding sample transferability should be considered. Participants involved in community groups were overrepresented, limiting in-depth exploration of the experiences of socially isolated individuals who may face greater adverse mental health outcomes. However, as the purpose of this qualitative study was to explore phenomena and processes in depth rather than to generate generalizable data, the findings offer valuable insights. Specifically, the experiences shared by the participants provide a deep understanding of mental health and care access, which are relevant to community-dwelling older adults who utilize primary care. Nevertheless, these results should not be generalized to older adults who are more isolated, severely ill, have mobility limitations, or are institutionalized. This population often experiences adverse health trajectories, including a higher likelihood of emergency department visits and hospitalization [64]. Furthermore, they may experience additional barriers that limit their access to mental health care in primary settings. Therefore, future research should focus on older adults who are not engaged in community groups, have less frequent contact with primary care, and experience greater health burdens. Similarly, this study was limited to residents of Santiago, the capital city. While Chile’s primary care mental health programs have national coverage, regional disparities in the distribution of symptoms of common mental disorders exist among older adults [65,66], which suggest structural and social regional differences that this study could not explore. Hence, these results should not be generalized to other sociocultural or economic contexts. Further research is necessary to deepen the understanding of older adults’ mental health care access across diverse settings in Chile.

## 5. Conclusions

Community-dwelling older adults in Chile who frequently use primary care face multidimensional facilitators and barriers to accessing mental healthcare. Strengthening these services requires integrating care, ensuring adequate funding, and enhancing team training to meet specific, life-stage needs. From a policy perspective, shifting from program availability to integrated, person-centered protocols is crucial.

## Figures and Tables

**Table 1:** Focus groups guide.

Topics Considered	Examples of Guiding Questions
Mental health of older adults and mental health care seeking	In your opinion, what does mental health in older age mean? What factors are involved in specific mental health problems such as depression in older age? In your view, when older adults experience depression or other mental health problems, how should they deal with it? In your view, what aspects make older adults with mental health needs seeking or not seeking mental healthcare?
Social and environmental factors involved in mental health care access	What do you think that other people, such as family members, neighbors, or health providers think about older adults who have mental health problems? What aspects of the family, community or society could have an impact on mental health of older adults?
Mental health care access in primary care	In your community, how is the process of accessing mental health care in primary care for older people experiencing mental health problems? Considering your own experience or the experience of older adults that you know, such as family members, friends, or neighbors, how is the experience of accessing and receiving mental health care in primary care? How should mental health care be organized and delivered to improve the access of older adults with mental health needs?

**Table 2:** Characteristics of participants.

**Variables**	**(n = 62)**
Age, median (min–max)	71.5 (62–88)
Gender, n (%)	
Male	25 (40.32)
Female	37 (59.68)
Years of education, n (%)	
<8	6 (9.68)
8–11	44 (70.97)
12+	12 (19.35)
Marital status, n (%)	
Married or with a couple	28 (45.16)
Single or divorced	20 (32.26)
Widowed	14 (22.58)
Depressive symptoms, n (%)	
None or mild	50 (80.65)
Moderate or severe	12 (19.35)

## Data Availability

The data analyzed in this study are not publicly available, since the transcripts contain information that may compromise participants’ confidentiality. De-identified data supporting the findings, such as summary charts, are available upon reasonable request.

## Supplementary Materials

The following supporting information can be downloaded at: https://www.mdpi.com/article/10.3390/healthcare14101341/s1, File S1: Consolidated criteria for reporting qualitative studies (COREQ): 32-item checklist.
