# Supplementary material for: Facilitators and Barriers to Accessing Mental Health Care for Older Adults Who Are Primary Care Users in Chile: A Focus Group Study
Source: Healthcare (Basel). 2026 May 14;14(10):1341. doi: 10.3390/healthcare14101341 (PMC13205244; doi:10.3390/healthcare14101341)
Supplement: Supplementary file 1 [file healthcare-14-01341-s001.zip › healthcare-4262869-supplementary.pdf]

Supplementary File 1

Consolidated criteria for reporting qualitative studies (COREQ): 32-item checklist

Item No	Guide Questions/Description	Reported on Page #
**Domain 1: Research team and reflexivity**		
**Personal Characteristics**		
1. Interviewer/ facilitator	Which author/s conducted the interview or focus group?	4
2. Credentials	What were the researcher's credentials? E.g., PhD, MD	6
3. Occupation	What was their occupation at the time of the study?	6
4. Gender	Was the researcher male or female?	6
5. Experience and training	What experience or training did the researcher have?	6
**Relationship with participants**		
6. Relationship established	Was a relationship established prior to study commencement?	6
7. Participant knowledge of the interviewer	What did the participants know about the researcher? e.g. personal goals, reasons for doing the research?	6
8. Interviewer characteristics	What characteristics were reported about the interviewer/facilitator? e.g. Bias, assumptions, reasons and interests in the research topic	6
**Domain 2: study design**		
**Theoretical framework**		
9. Methodological orientation and Theory	What methodological orientation was stated to underpin the study? e.g. grounded theory, discourse analysis, ethnography, phenomenology, content analysis	3
**Participant selection**		
10. Sampling	How were participants selected? e.g., purposive, convenience, consecutive, snowball	3,4
11. Method of approach	How were participants approached? e.g., face-to-face, telephone, mail, email	4
12. Sample size	How many participants were in the study?	6
13. Non-participation Setting	How many people refused to participate or dropped out? Reasons?	3,4
14. Setting of data collection	Where was the data collected? e.g., home, clinic, workplace	4
15. Presence of nonparticipants	Was anyone else present besides the participants and researchers?	4
16. Description of sample	What are the important characteristics of the sample? e.g. demographic data, date	3,6,7
**Data collection**		

Item No	Guide Questions/Description	Reported on Page #
17. Interview guide	Were questions, prompts, and guides provided by the authors? Was it pilot tested?	4,5
18. Repeat interviews	Were repeat interviews carried out? If yes, how many?	N/A
19. Audio/visual recording	Did the research use audio or visual recording to collect the data?	4
20. Field notes	Were field notes made during and/or after the interview or focus group?	4
21. Duration	What was the duration of the interviews or focus group?	5
22. Data saturation	Was data saturation discussed?	4
23. Transcripts returned	Were transcripts returned to participants for comment and/or correction?	N/A
**Domain 3: analysis and findings**		
**Data analysis**		
24. Number of data coders	How many data coders coded the data?	6
25. Description of the coding tree	Did the authors provide a description of the coding tree?	N/A
26. Derivation of themes	Were themes identified in advance or derived from the data?	5,6
27. Software	What software, if applicable, was used to manage the data?	6
28. Participant checking	Did participants provide feedback on the findings?	N/A
**Reporting**		
29. Quotations presented	Were participant quotations presented to illustrate the themes/findings? Was each quotation identified? e.g., participant number	8-10
30. Data and findings consistent	Was there consistency between the data presented and the findings?	8-10
31. Clarity of major themes	Were major themes clearly presented in the findings?	8-10
32. Clarity of minor themes	Is there a description of diverse cases or a discussion of minor themes?	8-10
